# Low‐grade BRAF V600E mutant oligodendroglioma‐like tumors of children may show EGFR and MET amplification

**DOI:** 10.1111/bpa.12904

**Published:** 2020-11-02

**Authors:** Rui Ryan Yang, Kay Ka‐Wai Li, Anthony P.Y. Liu, Hong Chen, Nellie Yuk‐Fei Chung, Aden K.Y. Chan, Fangcheng Li, Danny Tat‐Ming Chan, Ying Mao, Zhi‐Feng Shi, Ho‐Keung Ng

**Affiliations:** ^1^ Department of Neurosurgery Guangzhou Women and Children's Medical Center No. 9 Jinsui Road Guangzhou 510623 China; ^2^ Department of Anatomical and Cellular Pathology Prince of Wales Hospital The Chinese University of Hong Kong 30‐32 Ngan Shing Street Shatin Hong Kong China SAR; ^3^ Department of Paediatrics and Adolescent Medicine Queen Mary Hospital The University of Hong Kong Hong Kong China SAR; ^4^ Department of Pathology Huashan Hospital Fudan University Wulumuqi Zhong Road 12 Shanghai 200040 China; ^5^ Department of Neurosurgery Prince of Wales Hospital The Chinese University of Hong Kong 30‐32 Ngan Shing Street Shatin Hong Kong China SAR; ^6^ Department of Neurosurgery Huashan Hospital Fudan University Wulumuqi Zhong Road 12 Shanghai 200040 China

Pediatric low‐grade gliomas were shown to be characterized by an array of distinct molecular aberrations. The recent cIMPACT‐4 consensus proposed pediatric low‐grade gliomas of the diffuse type to be characterized by distinct molecular changes rather than distinct histological features (2). Very recently, Fukuoka *et al* described a small series of pediatric oligodendroglioma‐like tumors with BRAF V600E mutations (3). Interestingly, they exhibited molecular changes usually associated with adult high‐grade gliomas: chromosome instability, chromosome 7 gains and chromosome 10 loss, but had an indolent natural history.

We have collected four more cases which we believe have great similarity to Fukuoka *et al*’s cases.

## Patient 1

An 8‐year‐old boy presented to our hospital with on‐and‐off frontal headache for several months and found to have right optic disc swelling during routine eye check‐up in school. MRI 6 months later showed a 1.1 cm roundish lesion in right temporal lobe. Serial MRI scans showed enlargement of the lesion to 1.45 cm after 18 months (Figure S1A‐D). Craniotomy with gross total tumor excision was done. Histology showed an oligodendroglioma‐like tumor (Figure S1E). The patient had no neurological dysfunction postoperation. No adjuvant treatment was given. This was a very recent case and the patient was last followed up twelve months after operation. He was neurologically normal, attending school and there was no radiological recurrence.

## Patient 2

**Figure 1:**
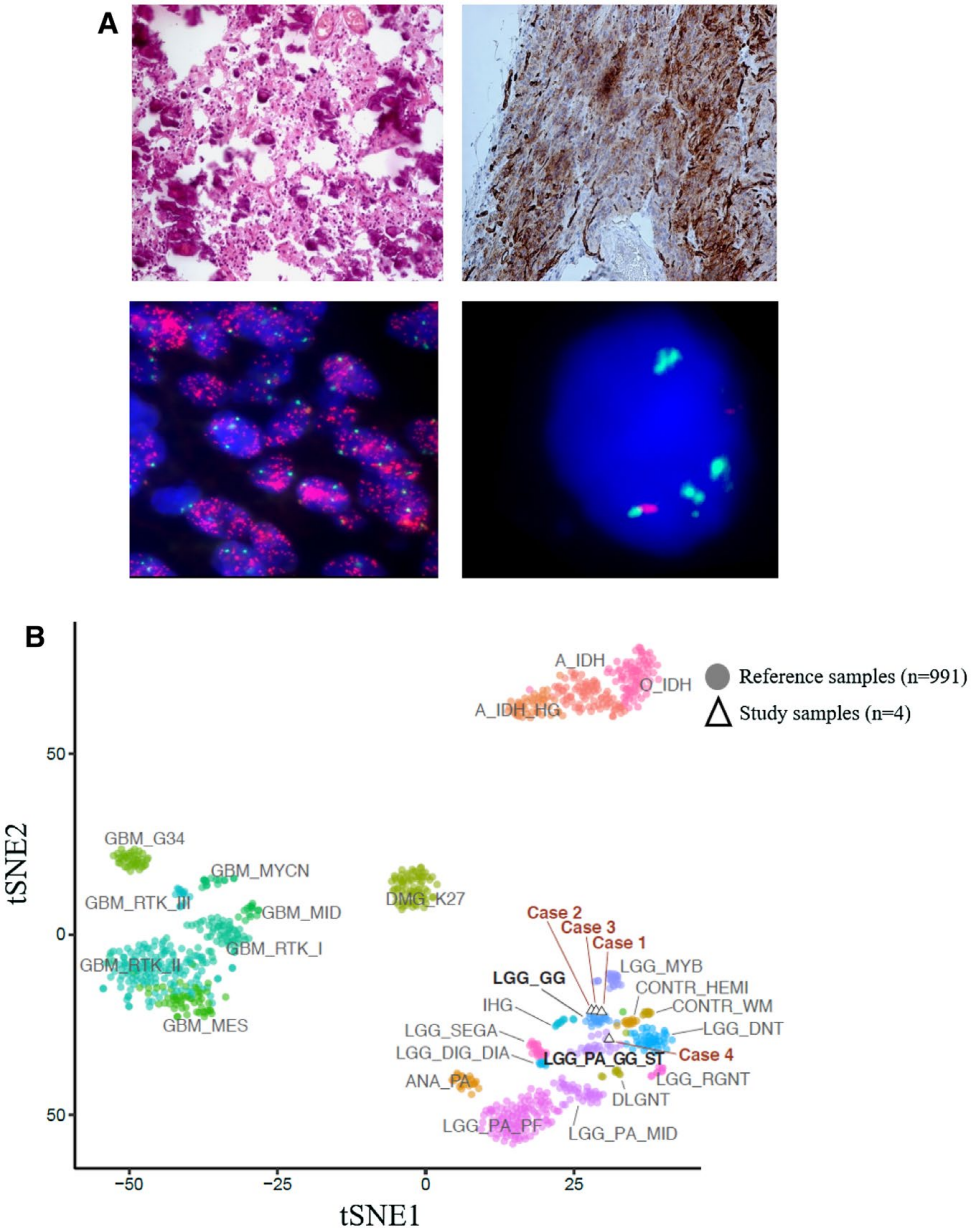
*Histology, immunohistochemistry profile and FISH finding of patient 2*. (**A**) Upper left, H&E shows an oligodendroglioma‐like tumor with heavy calcification. Upper right, CD34 shows strong cytoplasmic staining. Lower left, FISH assay for EGFR shows clusters of red signals in tumor nuclei. Target probe and reference probe are indicated as red and green respectively. Lower right, FISH assay for MET (Target probe: green; reference probe: red). (**B**) Methylation‐based t‐SNE plot of the four low‐grade BRAF V600E mutant oligodendroglioma‐like tumors of children and 911 well‐established reference samples. Reference cases are indicated as colored circles. Each reference methylation cluster is indicated by a unique color. Tumors in this study cohort are indicated as triangles and are labeled as cases 1–4.

A 12‐year‐old girl suffered from frequent partial complex seizures for one year before she was admitted into our hospital. MRI scan showed a small tumor at right temporal lobe close to middle cranial fossa. A gross total resection was achieved during surgery. Histological diagnosis was oligodendroglioma, Grade II (Figure 1A). Conventional radiotherapy but no chemotherapy was given. Patient now survived 162 months with no recurrence. She had a normal working life with no neurological deficit and was free of seizure.

## Patient 3

A 15‐year‐old girl suffered from sudden onset of grand mal seizure attack 5 days before admission. MRI scan showed left temporal lobe tumor and gross total resection was achieved. Histological diagnosis was oligodendroglioma (Figure S2A). Conventional postoperative chemo (temozolamide)‐radiotherapy was given. Patient had a normal life and went to university, finishing off with a degree and the overall survival time was 104 months.

## Patient 4

An 8‐year‐old boy was found to have a brain tumor incidentally by a CT scan after falling from height. A small tumor was found at the dorsal brain stem at the corpora quadrigemina area. Patient underwent craniotomy and gross total resection was achieved. Histological diagnosis was oligodendroglioma, Grade II (Figure S3A). After surgery, patient was prescribed carmustine. No radiotherapy was performed. There had been no recurrence during last follow‐up. Patient was alive and well 95 months after surgery.

Histologically, all four tumors were very similar and resembled closely adult oligodendroglioma: sheets of monomorphic cells with round nuclei and perinuclear halos and “chicken‐wire” pattern of branching capillaries. Microcalcification was present in tumors 1, 2 and 3 but was absent with tumor 4. High‐grade cytological features, mitosis, necrosis or endothelial proliferation were not found. No astrocytic or other glial component was present in all four tumors and no neuronal‐glial component typical of DNET was present. All tumors were positive for Olig2. CD34 was strongly stained in three cases, but was negative for case 4 (Figure 1A, Figures S1F, S2B, and S3B). Ki67 index was low for all four cases, and ranged between 1% and 2%.

Sanger sequencing confirmed the presence of BRAF V600E mutation in all four tumors (Figure S4). None of the samples carried C228T and C250T mutations of TERT promoter. All four tumors were negative for IDH1 and IDH2 mutations. All cases were subjected to methylation profiling by EPIC array. T‐SNE dimensionality reduction (Figure 1B) showed that overall our four tumors were clustered very well within the methylation clusters belonging to the “pediatric‐type” low‐grade gliomas. Three of our samples (75%) fell into the methylation cluster LGG_GG (methylation class low‐grade glioma, ganglioglioma) and they were in close proximity to each other. The remaining one sample fell into the methylation cluster LGG_PA_GG_ST (low‐grade glioma, subclass hemispheric pilocytic astrocytoma and ganglioglioma). Unsupervised clustering of DNA methylation patterns of our four samples alongside well‐characterized glioma‐related reference samples from the Capper *et al* study (1) also showed three of our tumors fell into the methylation cluster LGG_GG and the remaining one fell into the methylation cluster LGG_PA_GG_ST (Figure S5). We also submitted the genome‐wide DNA methylation profiling to DKFZ Classifier which revealed that two of our cases were classified as “no matching methylation classes” (patients 2 and 4). The other two cases were classified into methylation class low‐grade glioma, ganglioglioma with a calibrated score of 0.92 (patient 1) and 0.79 (patient 3).

Copy number variation was determined from the methylomes using the “conumee” R package. Recurrent arm‐level gain or amplification was found at chromosomes 5 (n = 3), 7 (n = 2), 8 (n = 2), 19 (n = 2) and 20 (n = 2) (Figure S6). Recurrent arm‐level loss was not found in this study. Copy number alterations for several establish relevant glioma‐associated genes, including CDKN2A, EGFR, MDM2, MDM4, MET, MYB, MYC, MYCN, PDGFRA, PTEN and TP53, were then evaluated. Recurrent numerical alterations were identified for EGFR (n = 4; 100%), MET (n = 3; 75%) and PTEN (n = 2; 50%). None of our cases showed CDKN2A homozygous deletion. FISH studies confirmed that EGFR amplification or copy number gain in all four cases (Figure 1A, Figures S1H, S2C, and S3C). Two cases showed EGFR clusters in >95% (case 2) and ~70% (case 3) of their nuclei by FISH, and these suggested the presence of high‐level amplification. In the other two cases, 42% (case 1) and 62% (case 4) of nuclei containing three or more signals for EGFR probe, suggesting the presence of EGFR gain. Low‐level amplification of MET was confirmed in three cases (Figure 1A, Figures S2D and S3D). Cases 2, 3 and 4 had 8–9% of their nuclei demonstrating target/reference ratio >2. None of the cases showed 1p19q codeletion in CNV analysis and this was confirmed by FISH.

Next, we calculated total percentage of copy number variation according to the reported methodology (6). We found the frequency of CNV in the four samples ranged between 0.01% and 16.8% with a mean of 7.5% (median 6.6%). We also examined for the presence of reported fusion genes found in pediatric gliomas by employing two NanoString‐based panels developed by Dr. Cythnia Hawkins (4, 7). The panels covered ALK/BRAF/FGFR/ROS1/NTRK/MET‐related fusion genes reported in low‐grade and high‐grade gliomas in children. We did not identify any reported fusion genes in our cohort.

Pediatric oligodendroglioma‐like tumors are very enigmatic as they do not have IDH mutation and 1p19q codeletion of adult oligodendroglioma, in spite of the fact that they may look exceedingly similar to the latter. All of Fukuoka *et al*’s and our cases were histologically oligodendroglioma‐like but were BRAF mutated. BRAF V600E mutation is normally found in pediatric pleomorphic xanthoastrocytoma, and a subset of pilocytic astrocytoma and ganglioglioma. In pediatric low‐grade gliomas, it is usually associated with other molecular alterations, especially CDKN2A deletion. Fukuoka *et al*’s cases also showed footprints of high‐grade adult gliomas, like chromosomes 7 gains and 10 loss and chromosomal instability. And all four tumors of this series showed EGFR amplification/gains and three of them also displayed low‐level MET amplification, which are also findings normally found in adult glioblastomas. The findings from both series reinforce the fact that pathological diagnosis must be based on a combined, integrated approach of both histological and molecular findings. This would avoid the pitfall of erroneously assigning a high‐grade diagnosis to these low‐grade pediatric tumors because of the unusual molecular findings. Both our cases and Fukuoka *et al*’s were mostly CD34 positive. CD34, however, can also be found in gangliogliomas, pleomorphic xanthoastrocytomas, the so‐called “long‐term epilepsy associated tumors” (LEATs), dysembryoplastic neuroepithelial tumors and very recently, polymorphous low‐grade neuroepithelial tumors of the young (PLNTY) (5). One case of Fukuoka *et al*’s was only patchily stained for CD34 and one of our cases was negative.

We suspect our cases and Fukuoka *et al*’s cases also probably fall within the overall spectrum of PLNTY (5). PLNTYs were described to be occurring predominantly but not exclusively in children with a strong association with seizure and predilection for the temporal lobes. PLNTYs were oligodendroglioma‐like but may also contain astrocytic components which may be pleomorphic. They might even contain quasi‐ependymomatous components. PLNTYs and our and Fukuoka *et al*’s cases also showed mostly CD34 positivity. For MAPK pathway activation, only 3/10 of Huse *et al*’s series showed BRAF V600E mutations but interestingly, some of their cases showed FGFR fusions which also activate the MAPK pathway. There were also cases in Huse *et al* series without BRAF V600E and FGFR fusion transcripts. Further studies of a bigger series of pediatric oligodendroglioma‐like tumors in our view may help clarify the nature and classification of this group of tumors and related tumors.

## Conflict of Interest

All authors declare that they have no conflict of interest.

## Author Contributions

*Study design:* Ho‐Keung Ng, Kay Ka‐Wai Li. *Material preparation:* Ho‐Keung Ng, Rui Ryan Yang, Hong Chen, Fangcheng Li, Danny Tat‐Ming Chan, Ying Mao. *Data collection:* Kay Ka‐Wai Li, Anthony P. Y. Liu, Nellie Yuk‐Fei Chung, Zhi‐Feng Shi. *Data analysis:* Kay Ka‐Wai Li, Anthony P. Y. Liu, Nellie Yuk‐Fei Chung, Zhi‐Feng Shi. *Data interpretation:* Kay Ka‐Wai Li, Anthony P. Y. Liu, Aden K. Y. Chan, Ho‐Keung Ng. *Manuscript preparation:* Kay Ka‐Wai Li., Ho‐Keung Ng. *Review and editing:* Kay Ka‐Wai Li, Ho‐Keung Ng.

## Supporting information

Fig S1‐S6
**Figure S1.** **Histology, immunohistochemistry profile and FISH finding of patient 1.** (**A** and **B**) T1 MR images. (**C**) Lesion shows mild enhancement on contrast‐enhanced T1‐weighted imaging. (**D**) T2/FLAIR image. (**E**) H&E shows an oligodendroglioma‐like tumor with calcification. (**F**) CD34 shows strong staining. Neu‐N and neurofilament stainings show normal brain tissue infiltrated by tumor (data not shown). (**G**) IHC with BRAF V600E mutant‐specific antibody stains positive. (**H**) FISH assay for EGFR illustrates copy number gain for EGFR. Red and green signals represent the target probe and reference probe, respectively.
**Figure S2.** **Histology, immunohistochemistry profile and FISH finding of patient 3.** (**A**) H&E shows oligodendroglioma‐like tumor with calcification and vascular arcades. (**B**) CD34 shows strong staining. (**C**) FISH assay for EGFR shows clusters of red signals. (**D**) FISH assay for MET. Target probe and reference probe are indicated as green and red, respectively.
**Figure S3.** **Histology, immunohistochemistry profile and FISH finding of patient 4.** (**A**) H&E shows a tumor with many vacuolated cells with vascular network. Calcification is not seen. (**B**) CD34 staining is negative. (**C**) FISH assay for EGFR. Red and green signals represent target probe and reference probe, respectively. (**D**) FISH assay for MET. Target probe is illustrated by green signals.
**Figure S4.** **Sequencing electropherogram section for BRAF V600E.** The red arrow indicates a T to A nucleotide change at amino acid position 600.
**Figure S5.** Unsupervised clustering of DNA methylation patterns of reference samples in Capper *et al* study and four of samples in this study using the 15 000 most variably methylated probes.
**Figure S6.** **Arm‐level copy number variations in samples of this study.** Chromosome amplification/gain (shown in red) and losses (shown in blue) were identified by EPIC array. The numbers on top of the graph represent chromosome number (1–22), and the numbers on the left indicate patient number.

## Data Availability

The unprocessed IDAT files of genome‐wide methylation profiling can be downloaded from http://www.acp.cuhk.edu.hk/hkng. Other data supporting the findings of this study are available from the authors upon request.

## References

[1] Capper D , Jones DTW , Sill M , Hovestadt V , Schrimpf D , Sturm D *et al* (2018) DNA methylation‐based classification of central nervous system tumours. Nature 555:469–474. 29539639 10.1038/nature26000 PMC6093218

[2] Ellison DW , Hawkins C , Jones DTW , Onar‐Thomas A , Pfister SM , Reifenberger G , Louis DN (2019) cIMPACT‐NOW update 4: diffuse gliomas characterized by MYB, MYBL1, or FGFR1 alterations or BRAF(V600E) mutation. Acta Neuropathol 137:683–687. 30848347 10.1007/s00401-019-01987-0

[3] Fukuoka K , Mamatjan Y , Ryall S , Komosa M , Bennett J , Zapotocky M *et al* (2020) BRAF V600E mutant oligodendroglioma‐like tumors with chromosomal instability in adolescents and young adults. Brain Pathol 30:515–523. 31630459 10.1111/bpa.12799 PMC8018140

[4] Guerreiro Stucklin AS , Ryall S , Fukuoka K , Zapotocky M , Lassaletta A , Li C *et al* (2019) Alterations in ALK/ROS1/NTRK/MET drive a group of infantile hemispheric gliomas. Nat Commun 10:4343. 31554817 10.1038/s41467-019-12187-5 PMC6761184

[5] Huse JT , Snuderl M , Jones DT , Brathwaite CD , Altman N , Lavi E *et al* (2017) Polymorphous low‐grade neuroepithelial tumor of the young (PLNTY): an epileptogenic neoplasm with oligodendroglioma‐like components, aberrant CD34 expression, and genetic alterations involving the MAP kinase pathway. Acta Neuropathol 133:417–429. 27812792 10.1007/s00401-016-1639-9 PMC5325850

[6] Mirchia K , Snuderl M , Galbraith K , Hatanpaa KJ , Walker JM , Richardson TE (2019) Establishing a prognostic threshold for total copy number variation within adult IDH‐mutant grade II/III astrocytomas. Acta Neuropathol Commun 7:121. 31349875 10.1186/s40478-019-0778-3 PMC6660955

[7] Ryall S , Arnoldo A , Krishnatry R , Mistry M , Khor K , Sheth J *et al* (2017) Multiplex detection of pediatric low‐grade glioma signature fusion transcripts and duplications using the NanoString nCounter system. J Neuropathol Exp Neurol 76:562–570. 28863456 10.1093/jnen/nlx042

