# Supplementary material for: Low‐grade BRAF V600E mutant oligodendroglioma‐like tumors of children may show EGFR and MET amplification
Source: Brain Pathol. 2020 Nov 2;31(1):211–4. doi: 10.1111/bpa.12904 (PMC8018073; doi:10.1111/bpa.12904)
Supplement: Supplementary file 1 — Fig S1‐S6 Figure S1. Histology, immunohistochemistry profile and FISH finding of patient 1. (A and B) T1 MR images. (C) Lesion shows mild enhancement on contrast‐enhanced T1‐weighted imaging. (D) T2/FLAIR image. (E) H&E shows an oligodendroglioma‐like tumor with calcification. (F) CD34 shows strong staining. Neu‐N and neurofilament stainings show normal brain tissue infiltrated by tumor (data not shown). (G) IHC with BRAF V600E mutant‐specific antibody stains positive. (H) FISH assay for EGFR illustrates copy number gain for EGFR. Red and green signals represent the target probe and reference probe, respectively. Figure S2. Histology, immunohistochemistry profile and FISH finding of patient 3. (A) H&E shows oligodendroglioma‐like tumor with calcification and vascular arcades. (B) CD34 shows strong staining. (C) FISH assay for EGFR shows clusters of red signals. (D) FISH assay for MET. Target probe and reference probe are indicated as green and red, respectively. Figure S3. Histology, immunohistochemistry profile and FISH finding of patient 4. (A) H&E shows a tumor with many vacuolated cells with vascular network. Calcification is not seen. (B) CD34 staining is negative. (C) FISH assay for EGFR. Red and green signals represent target probe and reference probe, respectively. (D) FISH assay for MET. Target probe is illustrated by green signals. Figure S4. Sequencing electropherogram section for BRAF V600E. The red arrow indicates a T to A nucleotide change at amino acid position 600. Figure S5. Unsupervised clustering of DNA methylation patterns of reference samples in Capper et al study and four of samples in this study using the 15 000 most variably methylated probes. Figure S6. Arm‐level copy number variations in samples of this study. Chromosome amplification/gain (shown in red) and losses (shown in blue) were identified by EPIC array. The numbers on top of the graph represent chromosome number (1–22), and the numbers on the left indicate patient number. [file BPA-31-211-s001.pptx]

## Slide 1
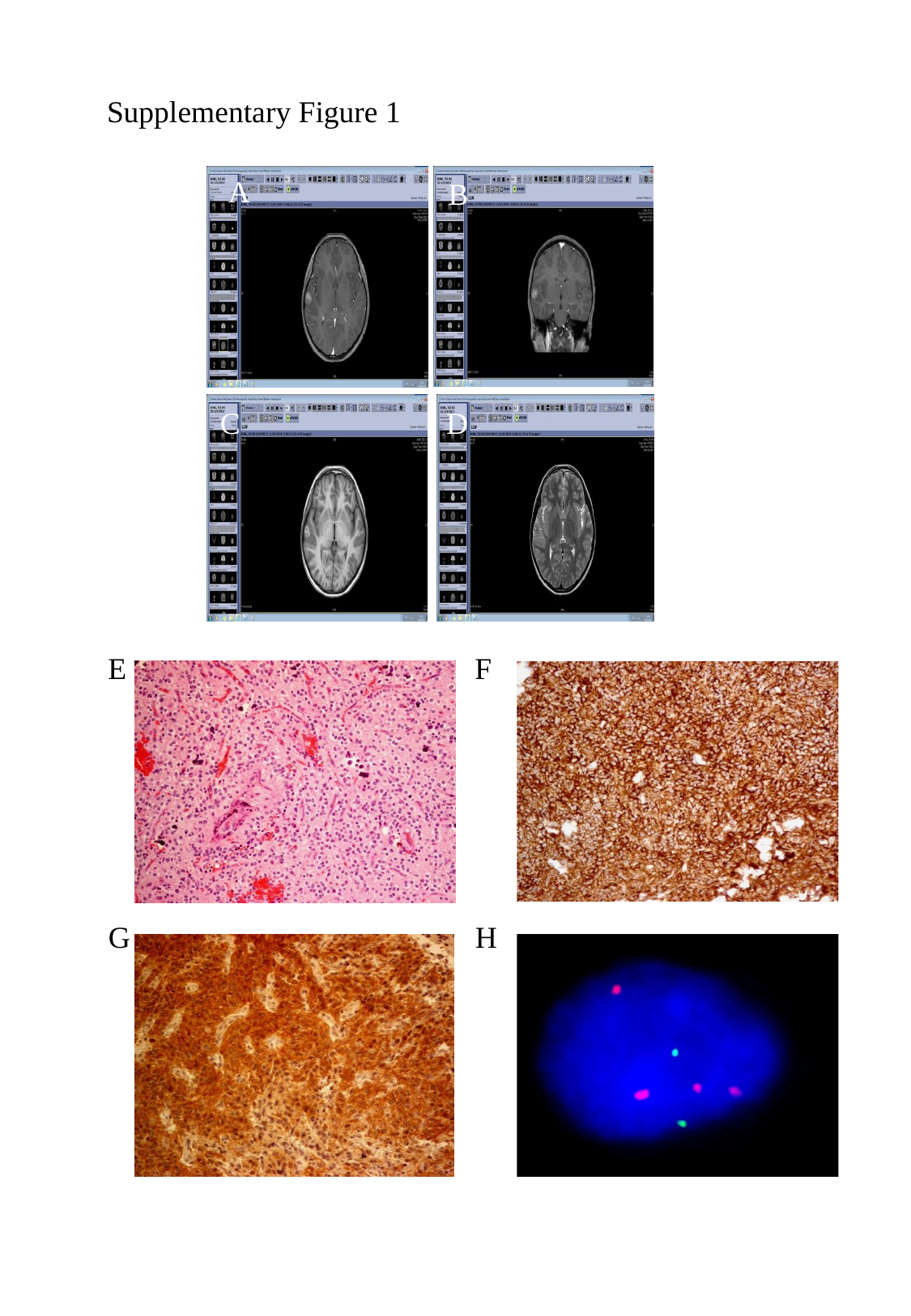

Supplementary Figure 1
A
B
C
D
E
F
G
H

## Slide 2
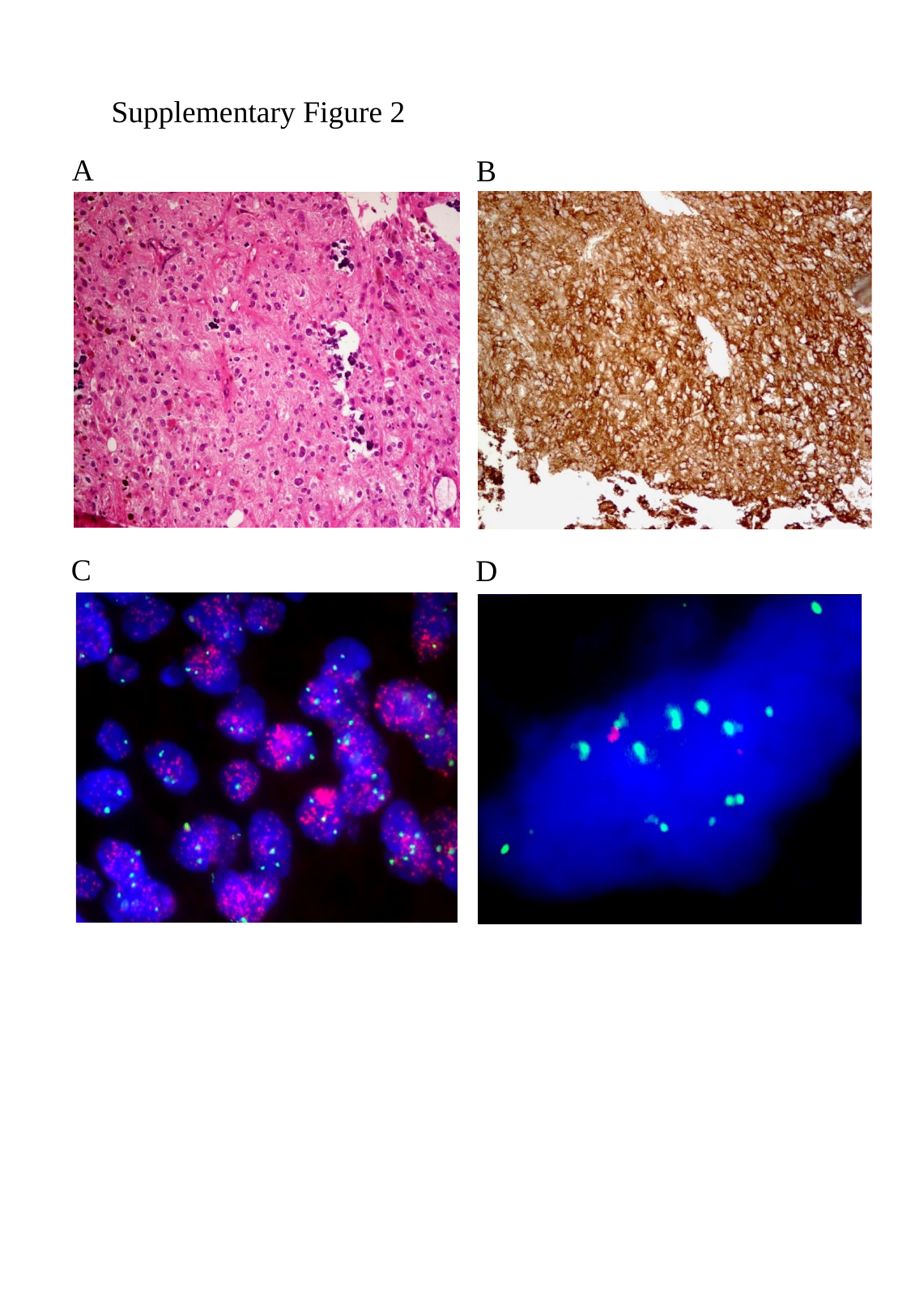

Supplementary Figure 2
A
B
C
D

## Slide 3
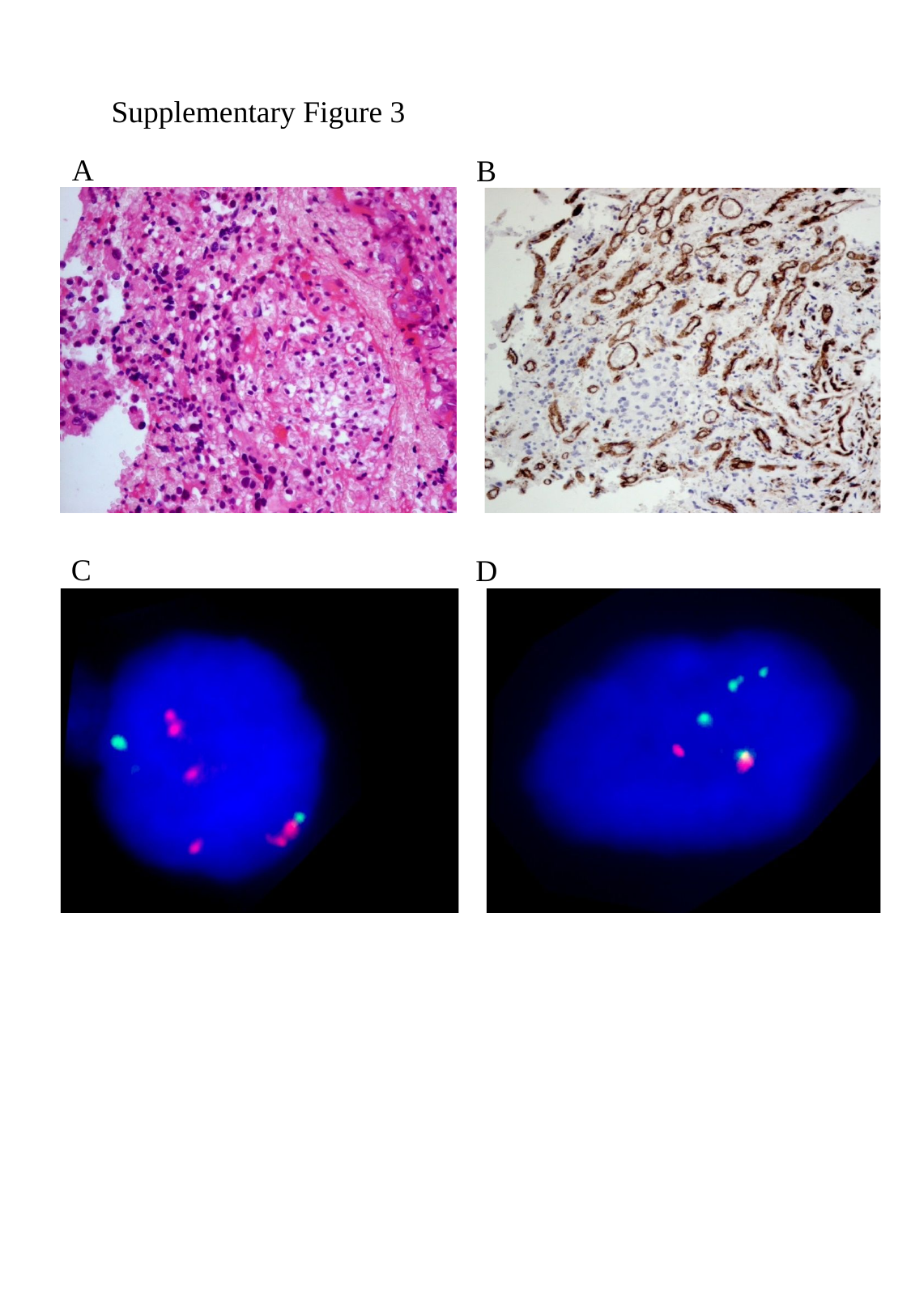

Supplementary Figure 3
A
B
C
D

## Slide 4
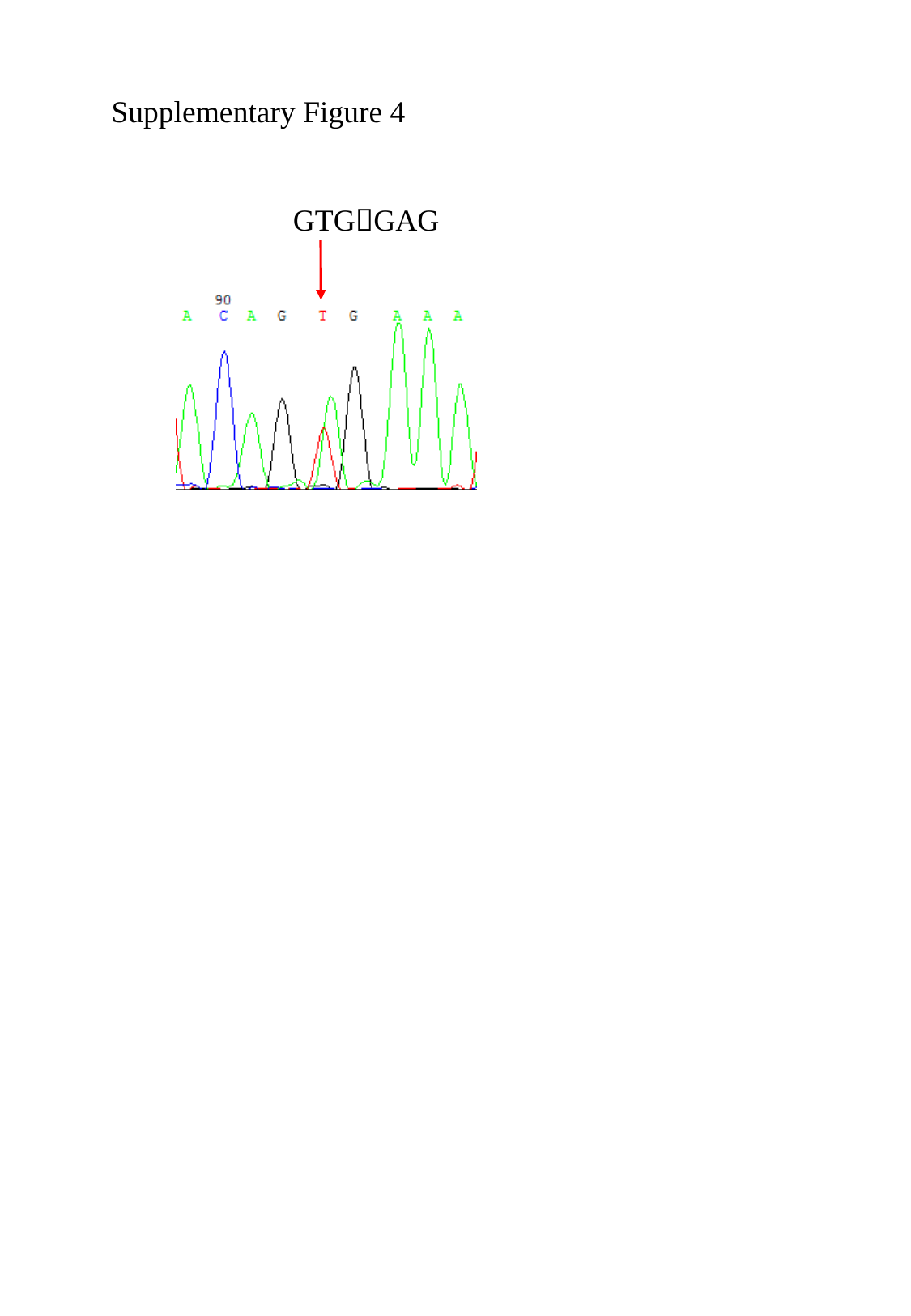

Supplementary Figure 4
GTGGAG

## Slide 5
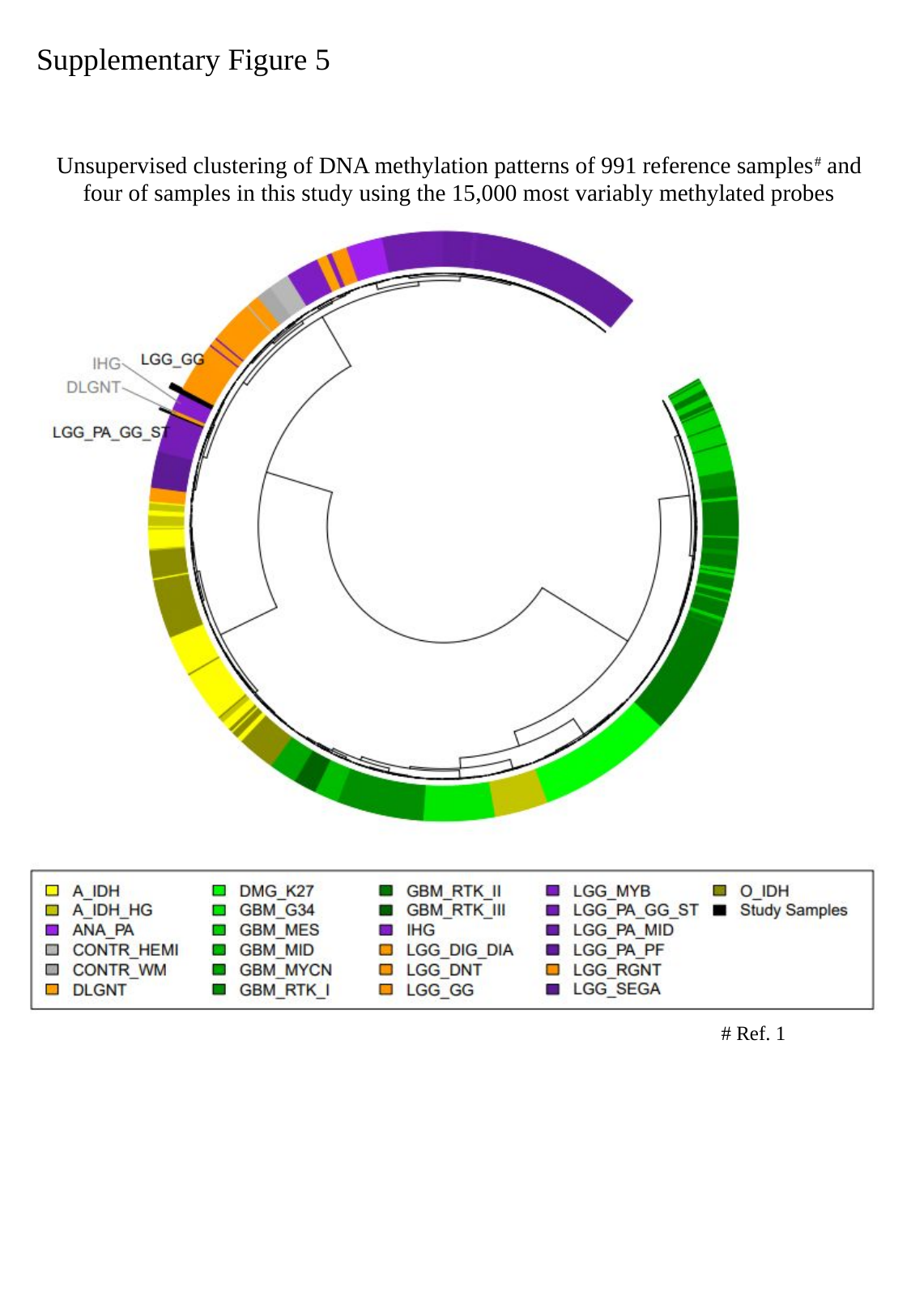

Supplementary Figure 5
Unsupervised clustering of DNA methylation patterns of 991 reference samples# and four of samples in this study using the 15,000 most variably methylated probes
# Ref. 1

## Slide 6
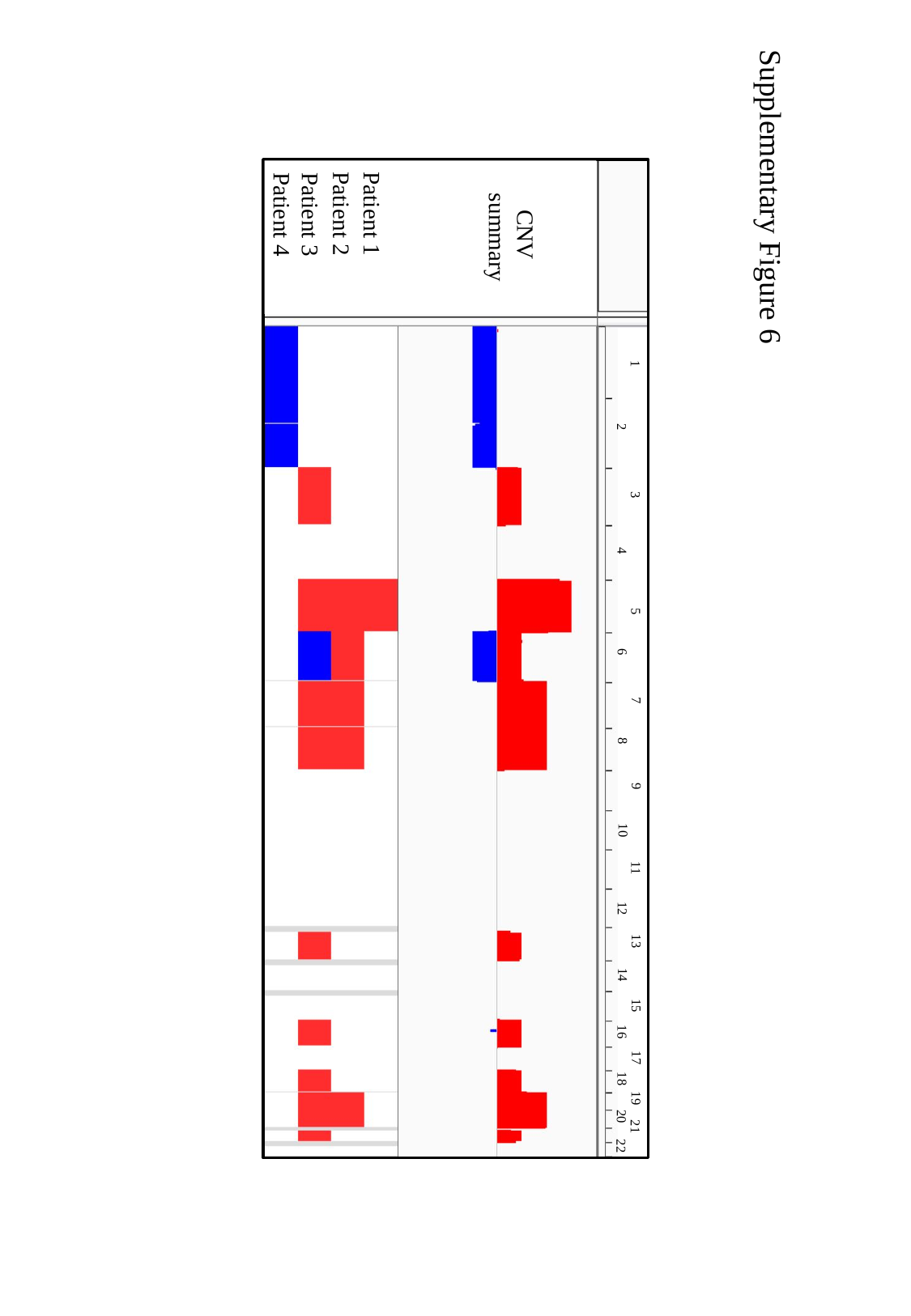

1
2
CNV
 summary
Patient 1
Patient 2
Patient 3
Patient 4
3
4
5
6
7
8
9
10
11
12
13
14
15
16
17
18
19
20
21
22
Supplementary Figure 6
